# Editorial for the Special Issue on Emerging Power Electronics Technologies for Sustainable Energy Conversion †

**DOI:** 10.3390/mi13040539

**Published:** 2022-03-30

**Authors:** Francisco J. Perez-Pinal

**Affiliations:** Tecnológico Nacional de México, Instituto Tecnológico de Celaya, Antonio García Cubas Pte. 600, Celaya 38010, Mexico; francisco.perez@itcelaya.edu.mx

Power electronic (PE) technology became considered a mature technology over the last century. Widespread silicon-based components and the emergence of modern modelling and control techniques have been the basis of the latest technological era.

Nevertheless, today we are living through a PE revolution, essentially motivated by three components: the arrival and application of wide-bandgap semiconductor technologies such as silicon-carbide, gallium-nitride and others in advanced power converter configurations; the appearance of improved modelling and control techniques such as non-integer philosophy to design fractional controllers; and the entrance of advanced ferromagnetic materials such as amorphous materials, nanocrystalline, powder cores, and Fe-Si alloys, to design magnetic components. The addition and combination of those three basic factors is predicted to be a feasible proposal to solve challenges related to cost, power consumption, power density and efficiency towards the development of small size and weight PE applications.

Accordingly, this Special Issue summaries nine research papers and one review on a wide range of power electronics technologies for sustainable energy conversion. Half of the manuscripts of this Special Issue [1,2,3,4,5] report applications of wide-bandgap and novel power electronic converters. Four of the papers [6,7,8,9] describe topics related to the modelling and control techniques of PE, and the remaining paper covers various aspects related to ferromagnetic materials.

In particular, Ma et al. [1] propose a distributed low voltage ride-through compensator (LVRTC) to be used in every wind turbine generator (WTG) for wind-farm applications. A step-by-step procedure to model and design the proposed controller using a dq-axis current decoupling algorithm and quantitative design of the three-phase inverter controller is given. Numerical results and hardware tests using a 2 kVA, six Silicon-Carbide switching device and a digital signal processor (DSP) as control core are reported. It is stated that the application of distributed LVRTCs increases reliability and flexibility, but decreases the required capacity and total price.

Following the same trend, the performance of an active neutral-point-clamped (ANPC) inverter operating with silicon (Si) and gallium trioxide (Ga_2_O_3_) devices is evaluated [2]. In this proposal, both devices are switched at different rates. The Si is operated at low frequency and the Ga_2_O_3_ is operated at high frequency. This switching configuration results in a homogeneously distributed power loss. Another interesting characteristic of the proposed arrangement is that the common mode current stress on the switches is reduced, a low common-mode voltage on the output is achieved and the fault current in the switching devices is mitigated. It is necessary to mention that the hybridization of ultra-wide bandgap (UWBG) semiconductor devices is still in development and its early stages. However, these promising results open the door to soon-achievable advances in this research direction.

On the other hand, a novel three-phase six-level multilevel inverter (MLI) controlled by a nearest-vector modulation strategy is reported in [3]. Numerical and practical results on a low-power test bed were reported. Advantages of the proposed MLI include a reduced number of devices, low total harmonic distortion (THD), and high efficiency (near 98%). The addition of the previous characteristic makes it suitable for high-voltage operations, utilization as an interconnected device in grid systems, and to be applied as a current and voltage compensation system, i.e., shunt active power filter (SAPF), dynamic voltage restorer (DVR) and unified power quality conditioner (UPQC).

Another interesting converter proposal was reported in [4]. In this manuscript, Loera-Palomo et al. proposed a novel non-cascade quadratic structure based on the interconnection of basic step-up and step-down configurations. The proposed converter has a noninverting output voltage and a minimum number of devices, and its quadratic property is valid only in continuous inductor conduction mode. A steady state (voltage and current source) boundary between continuous and discontinuous conduction mode, semiconductor stress analysis, dynamic model, numerical and experimental results are reported. In addition, a perturb and observe (P&O) maximum power point tracking was used to interconnect the reported system to a PV module. It is important to remark that the converter allows for a wide input voltage variation with a fixed output voltage, which is translated into a high-voltage conversion ratio property. This is a desirable characteristic in renewable energy applications where the energy source is variable.

Following this trend, in [5] a different converter with a quadratic voltage gain, common ground, and non-series power transfer is proposed. It consists of two traditional boost converters with a capacitor between both, which allows for parallel energy transmission between converters and output. The operation characteristics considering volt-second steady-state analysis, continuous conduction mode, ideal components, and pre-defined switching commutation, are reported. Additionally, a qualitative and practical quantitative comparison with other quadratic converters is also given, and bilinear and linear models of the proposed converter are also developed. The main advantage of this converter is that it increases the converter’s power efficiency due to the non-series power transfer, which was verified by a 500 W laboratory prototype. It is worth noting that the quadratic gain and practical high efficiency reported, above 95%, make this converter suitable for energy-harvesting systems.

Alternatively, a new resistive-inductance (RL) model for a proton-exchange membrane fuel cell (PEMFC) is reported in [6], which is compared with three well-known counterparts, i.e., nonlinear state-space, generic MATLAB™, and a resistive capacitance (RC). A step-by-step example to determine the proposed RL model’s parameters is also given. The four models were compared with static and dynamic responses of a practical 1.2 kW fuel-cell module; current–voltage graph, and time/voltage, respectively. Root-mean-square error (RMSE) was selected as the criterion during the static performance. On the contrary, for the dynamic performance, a load change of almost three times was applied and the different models’ performances were recorded. It was noticed that the non-linear model achieves the best functioning compared with the practical results, but it has the highest computational effort. Indeed, the proposed RL model achieves the second-best performance in both tests, but it is simple and accurate, which makes it a good candidate for real-time applications.

Following this scope, an all-inclusive conduction-mode independent (CMI) modelling for a boost converter is reported in [7]. This model is valid for the following cases: switched constant power (CPL), non-switched CPL, switched resistive load, and unswitched resistive load. A nonlinear controller, stability analysis based on Lyapunov, and numerical and experimental validation are also described for the CMI model with CPL. It is important to remark that, in contrast to traditional alternatives, the proposed controller does not rest on frequency control, and it is able to stabilize the boost converter with a CPL regardless of the operating conduction mode.

Another interesting manuscript is presented in [8]. Three pulse-width modulation methods derived from the traditional Space Vector PWM (SVPWM) technique were reported and applied to the transformerless photovoltaic DCM-232. This consists of a six-switch three-phase inverter plus four switches and two diodes that decouple photovoltaic supplies to generate two independent DC sources. The main aim of the proposed modulation methods was to control the decoupling switches to keep the common mode voltage constant, thereby achieving a leakage ground-current reduction. Numerical and practical results and a comparative efficiency analysis between the proposed PWM techniques and selected alternatives available in the literature were performed. It was concluded that all proposed modulation techniques were able to reduce the common mode current. As a result, the power inverter was able to fulfill international connection standards.

An advanced non-integer control strategy for a buck/boost converter was reported in [9]. To develop such kind of controller, the following steps were followed: an approximation, synthesis, and an implementation pathway. Here, the proposed non-linear synthesis is achieved through a biquadratic module that exhibits a flat phase response. Numerical and experimental results are provided to validate the usefulness of this approach. It is important to mention that implementation was carried by using analog operational amplifiers, which helped to achieve a fast practical response. However, the proposed non-integer approximated controller can also be extended to a digital version. To this end, traditional direct or indirect digital approximation can be used by using any sampled transformation.

Finally, a review of representative ferromagnetic core loss models is given in [10]. It includes amorphous, nanocrystalline powder cores and Fe-Si alloys. Characteristics, advantages, limitations, and applications of the more popular developed models reported in the literature are given. It is concluded that due to the non-linear features of ferromagnetic materials, the complexity of developing a unique power core loss model, which includes different ferromagnetic materials, and its respective validation, it is still incipient. Therefore, magnetic, and thermal loss calculus, modelling and ferromagnetic validation are still open research areas. The development of new ferromagnetic materials with specific features (core material, waveform, density flux, application, frequency range, among others) is still in the research and validation stages.

I would like to take this opportunity to thank all authors for submitting their manuscripts to this Special Issue. I would also like to acknowledge all reviewers for dedicating their time to providing careful and timely reviews to ensure the quality of this Special Issue.

## References

[1] Ma C.-T., Shi Z.-H. (2022). A Distributed Control Scheme Using SiC-Based Low Voltage Ride-through Compensator for Wind Turbine Generators. Micromachines.

[2] Meraj S.T., Yahaya N.Z., Hossain Lipu M.S., Islam J., Haw L.K., Hasan K., Miah M.S., Ansari S., Hussain A. (2021). A Hybrid Active Neutral Point Clamped Inverter Utilizing Si and Ga_2_O_3_ Semiconductors: Modelling and Performance Analysis. Micromachines.

[3] Meraj S.T., Yahaya N.Z., Hasan K., Hossain Lipu M.S., Masaoud A., Ali S.H.M., Hussain A., Othman M.M., Mumtaz F. (2021). Three-Phase Six-Level Multilevel Voltage Source Inverter: Modeling and Experimental Validation. Micromachines.

[4] Loera-Palomo R., Morales-Saldaña J.A., Rivero M., Álvarez-Macías C., Hernández-Jacobo C.A. (2021). Noncascading Quadratic Buck-Boost Converter for Photovoltaic Applications. Micromachines.

[5] Diaz-Saldierna L.H., Leyva-Ramos J. (2021). High Step-Up Converter Based on Non-Series Energy Transfer Structure for Renewable Power Applications. Micromachines.

[6] Belhaj F.Z., El Fadil H., El Idrissi Z., Intidam A., Koundi M., Giri F. (2021). New Equivalent Electrical Model of a Fuel Cell and Comparative Study of Several Existing Models with Experimental Data from the PEMFC Nexa 1200 W. Micromachines.

[7] Parada Salado J.G., Herrera Ramírez C.A., Soriano Sánchez A.G., Rodríguez Licea M.A. (2021). Nonlinear Stabilization Controller for the Boost Converter with a Constant Power Load in Both Continuous and Discontinuous Conduction Modes. Micromachines.

[8] Vazquez-Guzman G., Martinez-Rodriguez P.R., Sosa-Zuñiga J.M., Aztatzi-Pluma D., Langarica-Cordoba D., Saldivar B., Martínez-Méndez R. (2022). Hybrid PWM Techniques for a DCM-232 Three-Phase Transformerless Inverter with Reduced Leakage Ground Current. Micromachines.

[9] Sánchez A.G.S., Soto-Vega J., Tlelo-Cuautle E., Rodríguez-Licea M.A. (2021). Fractional-Order Approximation of PID Controller for Buck–Boost Converters. Micromachines.

[10] Rodriguez-Sotelo D., Rodriguez-Licea M.A., Araujo-Vargas I., Prado-Olivarez J., Barranco-Gutiérrez A.-I., Perez-Pinal F.J. (2022). Power Losses Models for Magnetic Cores: A Review. Micromachines.

